# A Case of Segmental Arterial Mediolysis Presenting as Mucosal Gastric Hematoma

**DOI:** 10.1155/2017/3634967

**Published:** 2017-11-23

**Authors:** Shunsuke Sakuraba, Hajime Orita, Shuhei Ueda, Satoshi Tokuda, Tomoaki Ito, Tomoyuki Kushida, Mutsumi Sakurada, Hiroshi Maekawa, Ryo Wada, Koichi Sato

**Affiliations:** ^1^Department of Surgery, Juntendo Shizuoka Hospital, Shizuoka, Japan; ^2^Department of Pathology, Juntendo Shizuoka Hospital, Shizuoka, Japan

## Abstract

**Background:**

Although segmental arterial mediolysis (SAM) has been increasingly recognized as arteriopathy and there are some case reports about SAM, it is still very rare. It is characterized clinically by aneurysm, dissection, stenosis, and occlusion within splanchnic arterial branches, causing intra-abdominal hemorrhage or bowel ischemia. Mortality is as high as 50% in acute events.

**Case Presentation:**

A 51-year-old man was referred to our hospital with hematemesis. Gastroscopy revealed a submucosal-like tumor on the posterior wall of gastric angle with ulceration. Computed tomography indicated a tumor measuring 65 × 50 mm in the stomach, which was suspected to have invaded into the pancreas. Significant hematemesis recurred; the patient developed shock and underwent emergency distal gastrectomy, distal pancreatectomy, and splenectomy. The pathology and the clinical course were compatible with SAM splenic artery rupture causing retroperitoneal hemorrhage that penetrated into the stomach. After that surgery, aneurysm of common hepatic artery ruptured and coil embolization was performed.

**Conclusion:**

SAM is an important cause of intra-abdominal or retroperitoneal hemorrhage in patients without underlying disease. SAM typically presents as intra-abdominal hemorrhage, but, in this case, the retroperitoneal hemorrhage penetrated into the stomach and it looked like a submucosal tumor.

## 1. Introduction

Segmental arterial mediolysis (SAM), previously called segmental mediolytic arteritis (SMA), is a rare nonarteriosclerotic, noninflammatory vascular disease first described in 1976 by Slavin and Gonzalez-Vitale [1–3]. Though reports about SAM have become much more frequent as knowledge about the disease and use of thin slice computed tomographic angiography have increased [4], the incidence is still only about 1 per 100,000 per year. The etiology is unknown but it is said that alpha-1 adrenergic receptors and beta-2 agonists such as ractopamine can cause similar condition. Ractopamine is used in many countries as a partitioning agent in pigs, but in Japan this agent is not permitted. A definite diagnosis is determined histologically and it is characterized by vacuolization and lysis of the outer media of arterial wall. Disease lesions typically occur in a skip pattern within splanchnic arterial branches, leading to aneurysm, dissection, stenosis, and occlusion. As an arteriopathy, SAM is similar to inflammatory vasculitis, collagen vascular disease, and fibromuscular dysplasia (FMD), so excluding these diseases is necessary. This disease is difficult to diagnose presymptomatically; therefore most cases present as spontaneous intra-abdominal hemorrhage, and a few cases appear as bowel ischemia, hematuria, or hemobilia. Particularly, bleeding sometimes causes a fatal result and may require urgent treatment. Coil embolization is currently well recognized as the first-line treatment whenever conservative therapy is not feasible, and the affected artery can be sacrificed. If embolization can cause distal ischemia or result in failure, surgical intervention is necessary.

In this case, we report a case of SAM with heterochronic vessel rupture of the splenic and common hepatic arteries, treated by surgical intervention and coil embolization.

## 2. Case Presentation

**Figure 1:**
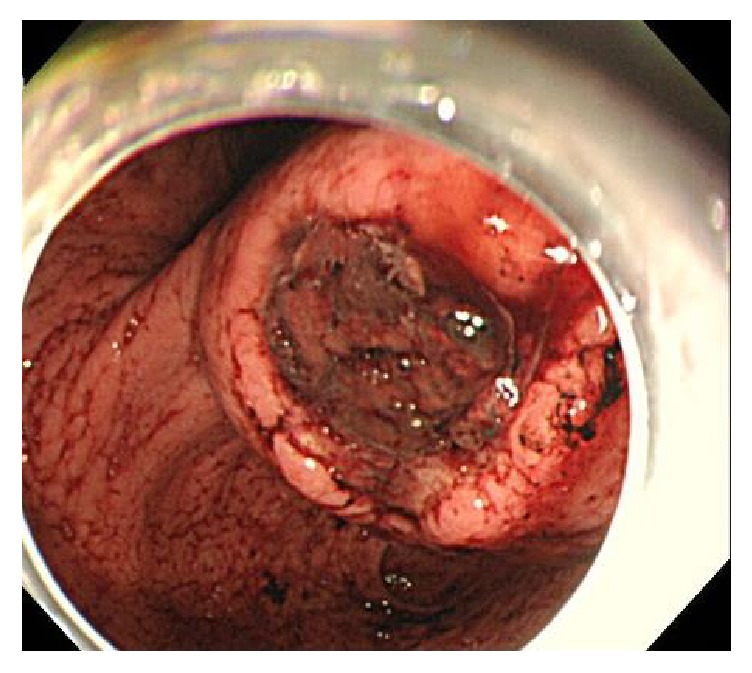
Gastroscopy revealed a submucosal-like tumor on the posterior wall of gastric angle with superficial ulceration.

**Figure 2:**
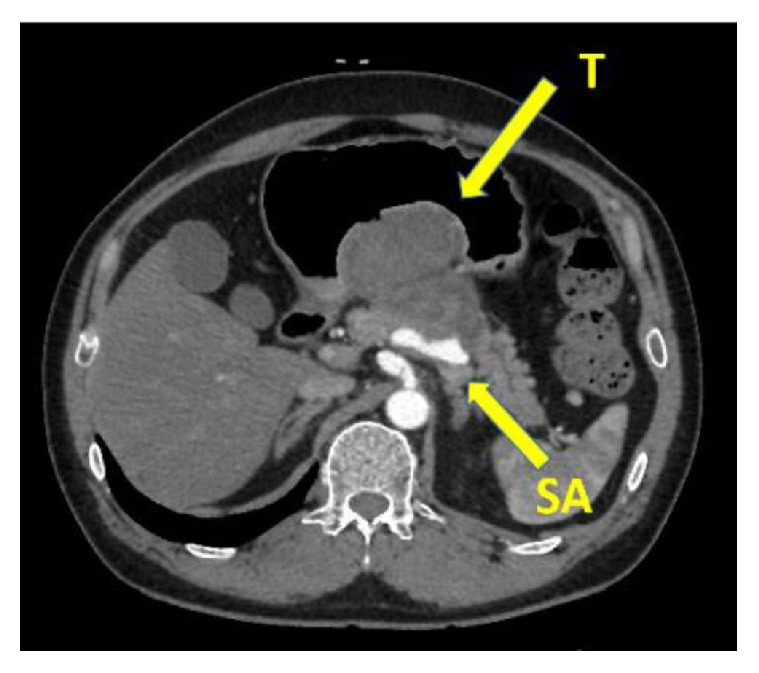
Computed tomography indicated a tumor measuring 65 × 50 mm in the stomach, which was suspected to have invaded into the pancreas, but no metastatic lesions were detected. T: tumor; SA: splenic.

**Figure 3:**
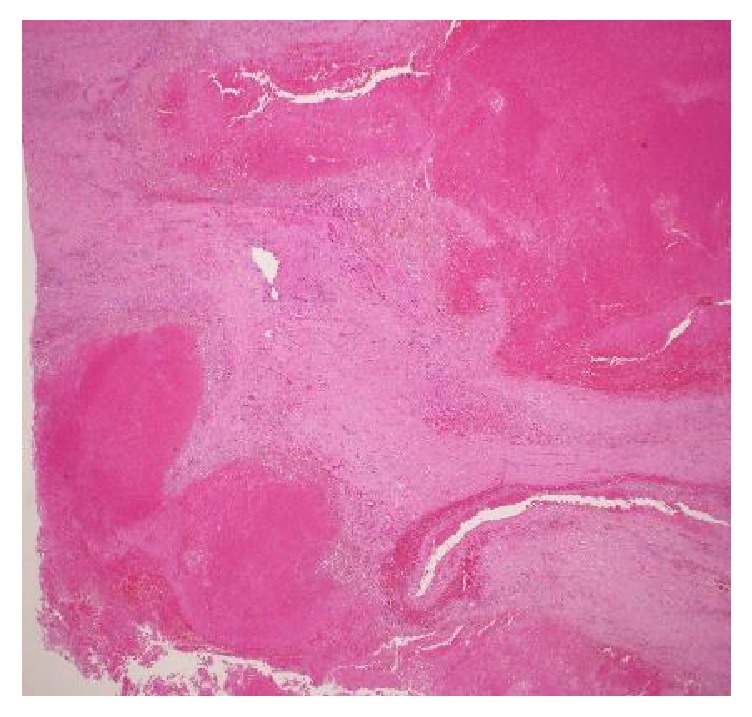
The resected specimen consisted mostly of a hematoma and some vessel wall.

**Figure 4:**
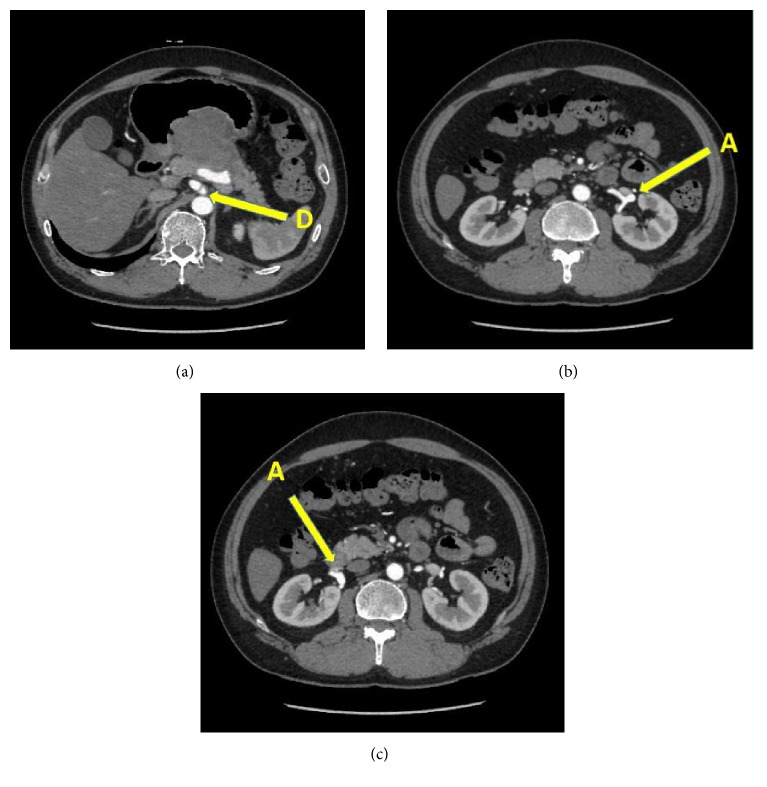
CT angiogram indicated dissection of common hepatic artery as well as aneurysms of the common hepatic artery (maximum diameter, 8 mm) and bilateral renal artery (maximum diameter, 5 mm). D: dissection; A: aneurysm.

The patient was a 51-year-old man who presented to the Emergency Department with hematemesis. His past medical history was unremarkable and he took no drugs. Physical examination revealed normal vital signs. On abdominal examination, the patient had no masses, muscle guarding, or rebound. Complete blood count revealed anemia with hemoglobin of 9.2. C-reactive protein (CRP) was normal, and the remainder of the serologic and immunologic workup was within normal limits. Gastroscopy revealed a submucosal-like tumor on the posterior wall of gastric angle with superficial ulceration (Figure 1). There were blood clots on top of the tumor, but no active bleeding existed; therefore we did not perform endoscopic hemostasis technique. Computed tomography indicated a tumor measuring 65 × 50 mm in the stomach, which was suspected to have invaded into the pancreas, but no metastatic lesions were detected (Figure 2). Magnetic resonance imaging also revealed a mass, having a high signal in the diffusion-weighted image (DWI) in the stomach, and the mass was also growing into the pancreas. At first, we considered the tumor as a malignant submucosal tumor such as GIST in the stomach or NET in the pancreas. General condition after hospital admission was stable, so we decided to perform an operation on suspicion of submucosal tumor, originating in the stomach or pancreas. During the preoperative period, massive hematemesis occurred with bright red blood with hemostatic shock. Emergency distal gastrectomy, distal pancreatectomy, and splenectomy were performed. The resected specimen consisted mostly of a hematoma and some vessel wall but did not have any malignant cells (Figure 3). We checked preoperative CT angiogram retrospectively and it indicated dissection of common hepatic artery as well as aneurysms of the common hepatic artery (maximum diameter, 8 mm) and bilateral renal artery (maximum diameter, 5 mm) (Figures 4(a), 4(b), and 4(c)). The tumor was close to splenic artery; therefore he was diagnosed with vessel rupture of splenic artery causing retroperitoneal hemorrhage and a hematoma that penetrated into the stomach.

**Figure 5:**
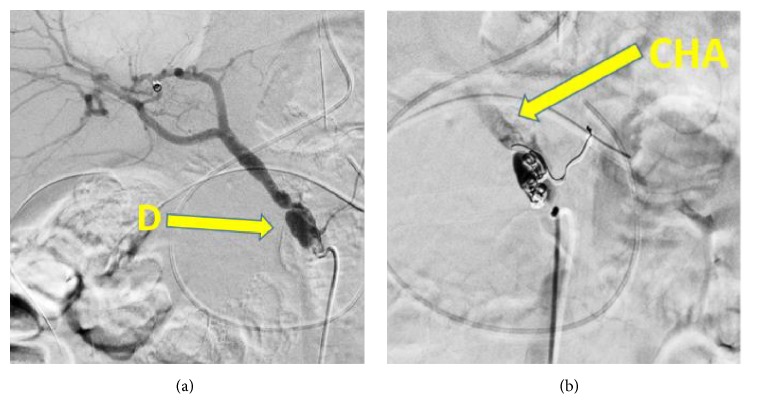
Coil embolization was performed for the aneurysm of common hepatic artery. D: dissection; CHA: common hepatic artery.

On postoperative day 21, he exhibited peritoneal irritation in the form of a rigid board-like abdomen with shock vital signs. Emergency CTA showed a dissection and vessel rupture of aneurysm of common hepatic artery, causing intra-abdominal hemorrhage, so he underwent emergency coil embolization (Figures 5(a) and 5(b)).

Follow-up was done every 6 months, and CTA one year after the first operation indicated the aneurysms (maximum diameter, 5 mm) of the bilateral renal arteries remained.

## 3. Discussion

SAM is a nonarteriosclerotic, noninflammatory vascular disease that commonly presents as spontaneous intra-abdominal hemorrhage from middle-size splanchnic arterial branches. Abdominal pain, anemia, and shock are the main symptoms. Some cases present as bowel ischemia, hematuria, and hemobilia, but this is rare. Bleeding from the splanchnic artery sometimes causes a fatal results, so prompt diagnosis and treatment are required. To diagnose SAM, computed tomographic angiography (CTA), especially thin slice CTA, is very helpful. Digital subtraction angiography (DSA) is also useful because when the lesion is identified, we can treat it with embolization. For radiographic features, CT scans typically show mesenteric or intraperitoneal hemorrhage, and CTA and arteriography features include fusiform aneurysms, stenosis, dissections, and occlusions within the splanchnic arteries. A pattern of aneurysms and stenosis in series is characteristic, the “string-of-beads” appearance. Pathology is the diagnostic gold standard, and the underlying histological process is lysis of the smooth muscle of the outer media of the arterial wall. In cases in which collecting a specimen is not viable, CTA helps us to discover SAM lesions and it is thought to be sufficient to diagnose SAM [5]. It affects the visceral arteries of the abdomen in a skip pattern, most commonly affecting the medium size branches of the superior mesenteric artery. Included in the differential diagnosis are inflammatory arteritis, collagen vascular disease, mycotic aneurysms, cystic medial necrosis seen in type IV Ehlers Danlos and Marfan's syndrome, and fibromuscular dysplasia (FMD) which are also important to consider [6, 7]. Laboratory markers of inflammation, differences in distribution, age, gender, and stigma may help to distinguish these diseases.

Typically, SAM presents in middle-aged or elderly patients with no underlying disease, and laboratory markers of inflammation or collagen disease are absent. Because some angiographic findings are similar to fibromuscular dysplasia (FMD) findings, SAM is considered by some researchers to be an early lesion, a variant of FMD [2, 8–10]. However, FMD occurs mainly in young to middle-aged women, and the pattern of arterial involvement is different [5, 11]. Based on the standard treatment for intra-abdominal hemorrhage, the course of treatment is mainly divided into two ways, embolization and surgical intervention. Because the etiology is not inflammatory or autoimmune, immunosuppressive agents are counterproductive. Treatment with embolization is the first choice but if it results in failure, surgical intervention, such as resection of the injured arteries or artery bypass, is necessary [11]. Preventive embolization for a lesion discovered incidentally may be recommended depending on the size of aneurysm. The prognosis if the acute event is adequately treated is good. With time, the lesions may resolve or remain unchanged [5, 12].

This case differs in that the CT scan showed dissection of the common hepatic artery and aneurysms of the common hepatic artery, splenic artery, and bilateral renal arteries. At first, the splenic artery ruptured, resulting in a retroperitoneal hematoma which penetrated into the stomach and masked as a submucosal tumor. Because the chief complaint was not typical intra-abdominal hemorrhage but hematemesis, at first we considered tumors such as GIST or NET.

SAM typically presents as intra-abdominal hemorrhage, but there are a few cases in which bleeding occurs into the bowel lumen. If we diagnosed SAM based on the CTA findings, the first-line treatment for the hematemesis would be coil embolization. We should keep in mind the possibility of SAM alternatively presenting as digestive tract hemorrhage, for which surgery is preferred.

## 4. Conclusion

SAM is an important cause of intra-abdominal or retroperitoneal hemorrhage in patients without underlying disease. SAM typically presents as intra-abdominal hemorrhage, but in this case, the retroperitoneal hemorrhage penetrated into the stomach and it looked like a submucosal tumor.
